# A Study of the Impact of Internet-Based Instruction Integrated Innovation Education on University Student Entrepreneurial Team Collaboration and Strategic Innovation

**DOI:** 10.3389/fpsyg.2020.01264

**Published:** 2020-06-26

**Authors:** Danqing Zhao, Huaqian Zhong, Yingli Wu, Qianfu Zhou

**Affiliations:** ^1^Academy of Art & Design, Shaoyang University, Shaoyang, China; ^2^Graduate School, Adamson University, Manila, Philippines; ^3^School of Mechano-Electronic Engineering, Xidian University, Xi’an, China; ^4^College of Economics and Management, Northeast Forestry University, Harbin, China; ^5^School of Management, Wuhan University of Technology, Wuhan, China

**Keywords:** student entrepreneurial team, internet, innovation education, collaboration, strategic innovation

## Abstract

With the advent of knowledge economy, the competition of comprehensive national power gradually has shifted to talent competition. The cultivation of innovative talents and the incubation of student entrepreneurial teams are the primary educational goals of universities, which is an important part of the national innovation system. University student entrepreneurial teams present the attribute of high technology and unique knowledge advantage. The persistent innovation of student entrepreneurial teams is attributed to knowledge playing a critical role. In particular, in the knowledge-based economic era, student entrepreneurial teams with speed, connection, and intangible value creation have transformed the partly labor-intensive model into a knowledge-intensive competition model. Taking university student entrepreneurial teams as the research object, a total of 500 copies of questionnaire were distributed and 386 valid copies were retrieved, with a retrieval rate of 77%. Through the questionnaire survey, students’ awareness, emotion, and will of innovation in innovation education are understood, and their cognition and intention of team cooperation and strategic innovation are investigated. The research results are concluded as follows: (1) Innovation education presents significantly positive correlations with collaboration. (2) Collaboration shows remarkably positive correlations with strategic innovation. (3) Innovation education reveals notably positive correlations with strategic innovation. According to the research result, suggestions are proposed, expecting that China’s university student entrepreneurial teams could acquire the advantage of technological innovation by applying the opportunities of broadband and wireless network infrastructure and developing innovative applications and entrepreneurial models.

## Introduction

The development of university students, being the reserves of human resources and the sources of knowledge innovation, is directly related to national development tactics and the construction of an innovative country. To enhance university students’ innovative entrepreneurial learning efficiency, the universities have adopted a series of strategies, of which student entrepreneurial teams based on academic societies are established. Universities, as an important part of the national innovation system, are responsible for the cultivation of innovative talents. Student entrepreneurial teams, as the effective carrier and form of talent development, are verified and prove their status and function. A lot of student entrepreneurial teams present satisfactory performance on technological innovation, knowledge lead, and knowledge transformation (Yanjie et al., 2019).

A student entrepreneurial team often presents certain knowledge advantage on certain domains, and more student entrepreneurial teams confirm such knowledge advantage as the core competency and concentrate on the development of various innovation learning and entrepreneurial activities. Under such a thinking mode, however, student entrepreneurial teams might lose the grasp of other domains that student entrepreneurial teams should seek for proper partners externally. Besides, partners are not simply in internal universities, but should cover external organizations of enterprises and investors. For this reason, student entrepreneurial teams, in addition to their capabilities, should seek external partners, with other student entrepreneurial teams and external enterprises and investors as a whole, to cope with challenges from external environments (Guo and Hu, 2014). With the development of technology, innovative entrepreneurship has become an inevitable ability for university students. To keep the knowledge advantage in the competitive development, student entrepreneurial teams should be ahead in technological innovation and create advantageous technology outcomes in order not to be eliminated from the market. Talent development is not simply an issue concerning students, but it also concerns universities. In this case, student entrepreneurial teams should be concerned about the changes in external environment to enhance the effectiveness and efficiency of knowledge innovation, create differential outcomes, and promote entrepreneurial performance (Wu and Song, 2019).

The strategy related to innovation is “to comprehensively promote technology and apply the internet, to enhance emerging businesses and product creation with knowledge value added, and to apply network technology to reduce costs for production, stock, and transaction to further create emerging markets.” Specific measures related to innovation were “to establish booming innovation and entrepreneurial mechanisms” and “to review current education systems, reinforce innovation, and cultivate re-learning ability” as long-term measures. “Internet + education” is a new revolution in the field of education. The application of internet reaches the barrier and boundary of education, which makes all elements in the field of education separated but united, concentrated but dispersed. Internet information storage is large, efficient, and fast, which can fragment and integrate valuable information, and enable people to share through electronic market, portal resources, forum resources, and personal resources (Yong, 2018).

As a result, the space and time connections in the field of education can be permanently expanded and can serve the subjects of education. From the perspective of learning, the internet is connected with education; regarding teaching, resource sharing and innovation represented by Open University and hybrid learning are realized; in terms of organizational ecology, the development of self-organization, open education, and other new business forms is formed as representative of education (Günther et al., 2019). To improve the innovation and entrepreneurship ability of college students and provide new direction and carrier for the innovation and entrepreneurship education mode, it is necessary to integrate the internet thinking mode into the innovation and entrepreneurship courses of colleges and universities, and guide, educate, and train college students. Internet education, with education as the theme, supplemented by internet tools and internet thinking, promotes the optimization of educational activities. Apparently, applying the internet to education and innovation education presents the importance on the development of student entrepreneurial teams’ strategic innovation. Therefore, the effect of applying the Internet to innovation education on student entrepreneurial teams’ collaboration and strategic innovation was studied, expecting that domestic student entrepreneurial teams could acquire the advantage of technological innovation by applying the opportunity of broadband and wireless network infrastructure as well as the development of innovative applications and entrepreneurial models (Wu et al., 2019).

## Literature Review

### Web-Based Instruction

In the era of the development of network information technology, the promotion and application of network teaching has become the main component of education informatization and also the embodiment of the reform of the new teaching mode of higher education. Network teaching platform is the platform of various teaching activities in network teaching. It can integrate all education and teaching activities to display on the platform, which is an efficient and convenient way for colleges and universities to hold network teaching activities. Halim et al. (2014) defined web-based instruction (WBI) as a hypermedia-centered teaching program that utilized the attributes and resources of the World Wide Web (WWW) for creating a meaningful learning environment, aiming to cultivate learners’ learning activities of automatic learning and continued learning. Therefore, web-based instruction referred to learners being able to autonomously utilize the rich resources on the Internet for constructing personal knowledge (Ritala et al., 2015). Chen et al. (2015) proposed that web-based instruction was “the learning method applying network transmission to acquire learning information and contents” (Malekifar et al., 2014), including information technology, transmission of various teaching contents, accumulation and management of learning processes and experiences, learning communities, as well as designers, providers, and domain experts of teaching contents. Yoon et al. (2015) defined e-learning as “any objects to achieve definite learning objectives through the delivery, facilitation, or mediation of electronic technologies”. Web-based instruction created the environment to enhance learning and support teaching with the characteristics and resources of the Internet and the interface of the World Wide Web. In addition to the promotion of network in school education, various industries provide the employees more and flexible learning opportunities through the development of computer network technology, helping web-based instruction to become a new trend (Tran and Pham, 2016).

### Innovation Education

Scholars generally believe that innovation driven is the power exerted in entrepreneurship, which is the necessary condition and power for entrepreneurs. Meanwhile, college students are the subjects of knowledge-based innovation and entrepreneurship activities, and their entrepreneurship is the source of innovation-driven energy. Higher education bears two important basic functions of research and development and education (Shen et al., 2019; Shen and Ho, 2020). First, higher education trains talents, so it shoulders the important task of education innovation talents. Second, innovation talents are the source power of knowledge innovation, promoting knowledge progress and research and development. Chan et al. (2015a) explained innovation education as the education with the cultivation of innovation spirit and innovation capability as the basic value orientation. Mario and Henar (2016) indicated that the core was based on education; to cope with the challenge of the knowledge-based economic era in the comprehensive quality education practice, it stressed on research and solving the problems of students’ innovation awareness, innovation spirit, and innovation capability in the cultivation of basic education. Acumen (2015) mentioned that the goal of innovation education was not to pursue “instilling,” “refreshing,” and “keeping up” in traditional education and continuing education, but to “ignite,” aiming to release human potential and stress on the combination of potential development with the cultivation of excellent psychological quality and positive life attitudes. Holgersson and Granstrand (2017) pointed out innovation quality as the concern of innovation education; such quality presented governance and maximal time appropriateness for modern and future talents. Fedyk et al. (2014) explained that the fulfillment of innovation goals was the cultivation of innovation talents and the effect of education at various stages, rather than the cultivation at a certain stage of education. In this case, innovation education was the integrated education as well as the education thought, education philosophy, and education practice implemented in the lifelong education process. Brautzsch et al. (2015) explained innovation education in that an organizational leader should have the creative idea and positively establish various education training for innovation, such as members co-participating in decision making, shaping communication situations without leaders, and inducing members’ innovation opportunities, to further create innovative and excellent organizational culture, enhance overall competitive advantage, and maintain the status of the organization.

Referring to Chan et al. (2015a), the following dimensions are used for measuring innovation education.

(1) Innovation Awareness: it refers to individual psychological intention to pursue new knowledge. Once such intention is stabilized, it would become individual spirit and culture.
(2) Innovation Emotion: it refers to psychological experience to pursue new knowledge. The constant reinforcement of such experience would be transformed into individual motivation and ideal.
(3) Innovation Will: it refers to self-consciousness to pursue new knowledge. The maintenance of such state would become individual habits and personality.

In the education of entrepreneurship and innovation, it is extremely necessary to cultivate students’ sense of teamwork and team entrepreneurship. Each member plays a different and corresponding role. As they have different knowledge, skills, and experience, the role they play is also different. Knowledge sharing among team members lays the foundation for the growth and development of entrepreneurial teams. Each member needs to coordinate their work by sharing resources and knowledge, and knowledge sharing is the duty and responsibility of each team member.

### Collaboration

Walters (2016) regarded collaboration as two or more independent groups jointly planning and executing relevant operation to acquire larger success than independent operation. Brouthers et al. (2015) simply defined collaboration as all participants positively making efforts for the commonly set goals. García-Sánchez et al. (2017) regarded collaboration as the ability of independent but relevant organizations acquiring resource sharing and satisfying most needs. Nissen et al. (2014) regarded collaboration as departments in an organization or organizations, based on mutual understanding and common vision, sharing resources to achieve common goals. Arvidsson (2014) considered collaboration as the closest cooperation model between partners that the members from different domains could contribute different but complementary skills to integrate team value and further create higher value. Jiang et al. (2015) defined partners as collaboration relationship that an enterprise could join buyers or sellers for reducing costs and make changes through sellers’ participation in new product development, delivery, and logistics management to develop partnership. Sandvik et al. (2014) indicated that partnership management for successful collaboration contains professional knowledge, favorable processes, common goals and motivation, and same points of view, which were essential for internal organization or between organizations. Chan et al. (2015a) indicated that collaboration could provide competitive advantages for the common development of all partners. Accordingly, collaboration was the key to create value that the increasing function and application of collaboration would enhance collaboration and integration to create larger value.

The research results of Jiang et al. (2015) were applied to the entrepreneurship practice of student entrepreneurial teams in this study, where information sharing, decision synchronization, and motivation congruence were used for measuring collaboration.

(1) Information Sharing: it refers to sharing frontier information in various professional domains and market feedback and determining strategy directions according to such information.
(2) Decision Synchronization: it refers to cooperation members of student entrepreneurial teams, enterprises, and investor participating in planning or decision-making of technological innovation and markets.
(3) Motivation Congruence: it refers to the degree of cooperation members of student entrepreneurial teams, enterprises, and investors sharing costs, risks, and results.

### Strategic Innovation

Wang et al. (2014) considered that strategic innovation was the definition of an organization re-conceptualizing businesses for the competition by breaking industrial gaming rules and thinking new methods, i.e., changing industrial law of competition. Different from strategic changes of an organization, strategic innovation stressed on placing organizational strategies at the industrial level that were different from current industrial competition. Chatterji and Fabrizio (2014) regarded strategic innovation as the intersection of innovation and strategic management that stressed on how to face environmental changes and opportunities. Reijsen et al. (2015) indicated that strategic innovation re-thought the ability of current business models for creating new value for customers’ competitors and new wealth for all shareholders to create wealth; in such a discontinued era, strategic innovation was the key. Bates and Khasawneh (2015) regarded strategic innovation as the integration of strategy and innovation and proposed a strategic innovation spirit that strategic innovation appeared on development and wealth creation. Lopez et al. (2016) indicated that strategic innovation utilized the new development of external environment for changing industrial dynamics to rebuild gaming rules. Chen et al. (2015) regarded strategic innovation as the comparison with competitors to re-define the customer groups and market position. Tangaraja et al. (2016) mentioned that strategic innovation was planned with further aspects and treated strategic innovation processes with multiple points of view to break through the boundary of current business models and think of innovation opportunities. Halim et al. (2015) considered that strategic innovation explored the basic definition of business through confirming potential customers, delivering customer value, and designing the structure of terminal value chain. Based on Yong et al. (2015), business model, change of competition rules, and value innovation are developed for measuring strategic innovation.

### Effects of Innovation Education on Collaboration

Mario and Henar (2016) indicated that an organization intending to create major innovation should apply innovation education so that the members would more conveniently conceive new ideas. Innovation education introduced the idea of collaboration to an organization to become the core structure of innovation programs; meanwhile, customers and colleagues should be invited to co-participate in the entire idea conceiving process (Brautzsch et al., 2015) and to find out more effective new methods and allow the members to apply emerging telecommunication technology so that the organization could conceive extremely creative ideas. Jiang et al. (2015) indicated that innovation education could have an organization reinforce the decision making and good performance through information exchange and sharing among collaborative support groups. The following hypothesis is therefore proposed in this study.

H1: Innovation education presents significantly positive correlations with collaboration.

### Effects of Collaboration on Strategic Innovation

Tangaraja et al. (2016) considered that collaboration could enhance knowledge sharing, including explicit and tacit knowledge, to enhance knowledge creation and innovation diffusion, to improve communication and coordination capabilities through collaboration, jointly solve problems, and reduce purchase costs with transactional contrast costs. In this case, student entrepreneurial teams with high collaboration would present better innovation capability, lower purchase costs, and better financial performance. Holgersson and Granstrand (2017) stated that enterprises could form competitive advantages through internal resources and capabilities as well as external corporate resources, which mainly came from the strategy network between organizations. Regarding strategic cooperation, Lopez et al. (2016) and Chen (2019) proposed to measure collaborative value innovation, where information sharing referred to value innovation-related information, including real-time sharing external trend to dig out business opportunities and the benefits and effectiveness of sharing products with customers (Yong et al., 2015; Liu et al., 2019). Decision synchronization referred to jointly planning and commonly making decisions for value innovation-related activities; collaborative businesses and investors could, through decision synchronization, avoid possible impact on the business model caused by external trend or re-define product function and position aiming at customer’s perceived product benefits and functions (Arvidsson, 2014). Accordingly, the following hypothesis is proposed in this study.

H2: Collaboration shows remarkably positive correlations with strategic innovation.

### Effects of Innovation Education on Strategic Innovation

Chan et al. (2015b) pointed out innovation as the law of survival of enterprises nowadays; however, innovation was an “idea,” which could be easily imitated, and an innovation strategy merely showed the advantage for 5 years. Chan et al. (2015b) stated that, for strategic innovation, a student entrepreneurial team should apply innovation education to adjust the thinking model of decision-making team; the traditional thinking of imitation, improvement, or pursuit of a single benchmark could not get out of the preset axis and hence it should be eliminated; a problem-solving model directly starting from market opportunities and business challenge could not excel and thus it should be adjusted. Chatterji and Fabrizio (2014) indicated that, for strategic innovation, a decision-making team should precede innovation education to present unprecedented originality for unexpected outputs. Yong et al. (2015) mentioned that the creativity of strategic innovation could be promoted through observation, understanding, imagination, association, and combination, which could be cultivated with innovation education. It is academically proven that creativity could be trained or induced; for student entrepreneurial teams, open mind, divergent thinking, and bravery to try might be the most important. As a result, the following hypothesis is proposed in this study.

H3: Innovation education reveals notably positive correlations with strategic innovation.

## Sample and Measuring Indicators

### Research Sample and Object

In this study, through the questionnaire survey, students’ awareness, emotion, and will of innovation in the innovation education are understood, and students’ cognition and intention of team cooperation and strategic innovation are investigated. This questionnaire mainly involves the following aspects: first, college students’ cognition of innovation activities; second, college students’ participation in innovation activities; and third, cultivation of students’ innovation awareness in innovation activities. In the aspect of innovation cognition, first, it investigates the importance of innovation activities in the eyes of students, which is divided into four topics: the enthusiasm of participating in activities, the different types of activities, the level of participating in innovation activities, and the influence of innovation activity credits on students’ enthusiasm. The current situation of school innovation activities is divided into four topics in the questionnaire: the satisfaction of innovation activities in the eyes of students, the way of students learning innovation activities, the interval time of innovation activities, and the form of students participating in innovation activities. The last three topics are about the cultivation of innovation awareness: first, college students’ awareness of innovation; second, whether the innovation awareness is improved after participating in innovation activities; and third, in which aspect the innovation awareness is improved.

In this study, the student entrepreneurial teams in Shaoyang University and Northeast Forestry University were selected as the research object. They were given a total of 500 copies of questionnaire, and 386 valid copies were recovered, with a retrieval rate of 77%. Shaoyang University is the education base for the practice of university students’ innovative entrepreneurship in China. It has established about 1,000 student entrepreneurial teams by relying on academic societies, and supports intercollegiate and interdisciplinary student entrepreneurial teams such that it is full of innovative entrepreneurship. Northeast Forestry University has established “Northeast Forestry University student innovative entrepreneurship education center” as the specific research and practice institution for the school developing university student innovative entrepreneurship education; takes charge of innovative entrepreneurship curriculum, innovative entrepreneurship practice base construction, entrepreneurial policy support, and entrepreneurship guidance and service; and is one of 50 innovative entrepreneurship universities in China. In this study, 386 samples were analyzed by SPSS26.0 software, and inappropriate items were modified or deleted to ensure the reliability and validity of each specific index in the questionnaire.

### Reliability and Validity Test

Validity refers to the measuring tool being able to really measure the problems that researchers intend to measure. Validity is generally divided into content validity, criterion-related validity, and construct validity. The questionnaire items in this study are referred to domestic and international researchers and a pretest is preceded before the distribution of formal questionnaire that the questionnaire presents certain content validity. Dimensions of innovation education, collaboration, and strategic innovation are tested, and the casual relationship with a linear structural relation model and the data entry is based on the correlation coefficient matrix of observation variables. The linear structural relation model analysis results reveal that the overall model fit reaches reasonable range. It therefore presents favorable convergent validity and predictive validity.

To further understand the reliability and validity of the questionnaire, both reliability and validity were analyzed. The higher Cronbach’s α reveals better reliability. The formal questionnaire was formulated according to the standard, and the measured Cronbach’s α was in 0.72–0.90, apparently conforming to the reliability range.

## Analysis of Empirical Results

The LISREL (linear structural relation) model combines factor analysis in traditional statistics and simultaneous equation in econometrics and could calculate multiple factors and multiple paths. The model fit could be evaluated with preliminary fit criteria, overall model fit, and fit of internal structure of model. The estimation process of the LISREL model is based on causality, which helps users of the model make deeper exploration, understand the problems studied, and deepen further thinking on causality. Although the path analysis method can be used to calculate the significant effect, it cannot be used in the case of many hidden variables. Hence, compared with the LISREL model, many assumptions are needed in path analysis, which requires relevant data and that data must be sufficient; otherwise, they cannot be applied. However, the LISREL model has relatively low requirements in this respect, and its scope of application is more extensive (Chen and Shen, 2020).

### LISREL Model Assessment Index

Item-to-total correlation coefficients are used for testing the construct validity of the questionnaire content in this study, i.e., reliability analysis. The calculated item-to-total correlation coefficients are used for judging the questionnaire content. The item-to-total correlation coefficients of the dimensions in this study are higher than 0.7, revealing a certain degree of construct validity of the dimensions.

The research data are given in Table 1, and the overall model fit is explained below. The overall model fit standards χ^2^/df = 1.637, lower than the standard 3, and RMR = 0.003, revealing the appropriate results of χ^2^/df and RMR. Furthermore, chi-square is extremely sensitive to sample size that it is not suitable for directly judging the fit. However, the overall model fit standards GFI = 0.976 and AGFI = 0.915 are higher than the standard 0.9 (the closer GFI and AGFI to 1, the better the model fit) that the model presents better fit indices.

**TABLE 1:** Analysis of overall model fit.

**Evaluation Item**	**Parameter/Evaluation Standard**	**Result**		***t***
Overall model fit	χ^2^/df	1.637		
	GFI	0.976		
	AGFI	0.915		
	RMR	0.003		

### Analysis of Preliminary Fit Criteria

The research data are organized in Table 2. From Table 1, the dimensions of innovation education (innovation awareness, innovation emotion, and innovation will) could significantly explain innovation education (*t* > 1.96, *P* < 0.05), the dimensions of collaboration (information sharing, decision synchronization, and motivation congruence) could remarkably explain collaboration (*t* > 1.96, *P* < 0.05), and three dimensions of strategic innovation (business model, change of competition rules, and value innovation) could notably explain strategic innovation (*t* > 1.96, *P* < 0.05). Apparently, the overall model presents favorable preliminary fit criteria.

**TABLE 2:** Analysis of preliminary fit criteria.

**Evaluation item**	**Parameter/evaluation standard**		**Result**	***t***
Preliminary fit criteria	Innovation education	Innovation awareness	0.706	11.22**
		Innovation emotion	0.715	12.38*
		Innovation will	0.694	10.23*
	Collaboration	Information sharing	0.683	9.16*
		Decision synchronization	0.675	8.74*
		Motivation congruence	0.699	10.86*
	Strategic innovation	Business model	0.712	11.94*
		Change of competition rules	0.723	13.12*
		Value innovation	0.735	14.51*

*p < 0.05, **p < 0.01.

### Analysis of Fit of Internal Structure of Model

Based on the research data in Table 3, innovation education shows positive and significant correlations with collaboration (0.837, *P* < 0.01), collaboration reveals positive and remarkable correlations with strategic innovation (0.866, *P* < 0.01), and innovation education presents positive and notable correlations with strategic innovation (0.874, *P* < 0.01) such that H1, H2, and H3 are supported. This shows that the innovation education in colleges and universities can cultivate students’ sense of team cooperation, and the sense of team cooperation can spread students’ strategic innovation thinking, which promotes improving the overall effect of innovation education.

**TABLE 3:** Analysis of fit of internal structure of model.

**Evaluation item**	**Parameter/evaluation standard**		**Result**	***t***
Fit of internal structure of model	Innovation education → collaboration		0.837	18.45*
	Collaboration → strategic innovation		0.866	22.61*
	Innovation Education → strategic innovation		0.874	24.53*

**Research hypothesis**	**Correlation**	**Empirical result**	***P***	**Result**

H1	+	0.837	*P* < 0.01	Supported
H2	+	0.866	*P* < 0.01	Supported
H3	+	0.874	*P* < 0.01	Supported

* P < 0.01.

According to the above fitting analysis results, combined with the current situation of innovation education in China, it is found that, at present, China has not yet formed a widely recognized collaborative culture. Due to the lack of collaborative innovation cooperation with spiritual core, it is difficult to form a long-term symbiotic development mechanism. University researchers mainly include teachers and students, who are usually combined in the form of innovative teams. Because the barriers between universities, and between universities and scientific research institutes have not been completely broken, there are still many difficulties in the sharing of human resources between them. In terms of scientific facilities sharing, scientific equipment resources refer to the places, instruments, and equipment provided by universities, enterprises, scientific research institutes, or government agencies for cooperative innovation. At present, the government formulates corresponding laws and regulations or rules for the resource sharing of scientific equipment in collaborative innovation. Hence, in this case, it is difficult for colleges, enterprises, and scientific research institutes to ensure the maximum open sharing of instruments, equipment, and laboratories, thus realizing the maximum utilization of resources.

## Conclusion

The research results reveal that the unique resources of student entrepreneurial teams are the possible source of the competitive advantage that student entrepreneurial teams could form long-term and continuous competitive advantages through the accumulation and cultivation of resources and capabilities. Most entrepreneurship programs of university student entrepreneurial teams are high-tech industries, which are technology-intensive knowledge industries; high-tech industries should apply innovation education to cultivate an innovative thinking model and originality as well as use the core assets and core competency for constructing the strategic innovation to grasp key values, create Blue Ocean, and accumulate more core resources. Introducing innovation education to student entrepreneurial teams with high-tech industries as the background would affect the collaboration. Such types of student entrepreneurial teams should be permanently concerned about the development of innovation education complemented with web-based instruction. The integration of web-based instruction into innovation education would introduce the idea of collaboration to an organization to become the core structure of innovation programs. Under such a situation, a student entrepreneurial team would attract other student entrepreneurial teams, enterprises, and investors more easily for the cooperation to gain strength, generate a benign loop, and further enhance the competitiveness. What’s more, favorable collaboration among student entrepreneurial teams and with enterprises and investors allows the university and university entrepreneurial teams to present more satisfactory performance, with the purpose of enhancing the innovative entrepreneurship education performance. The cooperation among student entrepreneurial teams and enterprises and investors could help the execution of strategic innovation. A student entrepreneurial team should conclude and maintain strong network relationship with strategic partners who could provide complementary resources and capabilities, share new technology and knowledge with partners, or achieve the goal of strategic innovation through strategic alliance so as to get rid of traditional market competition and acquire better entrepreneurship outcome.

This study confirms the positive correlation among innovation education, team cooperation, and strategic innovation, which contributes to the direction and mode of innovation education in colleges and universities. However, due to the limitations of personal ability, the questionnaire design of this study is not perfect, so the survey is not comprehensive. Next, the questionnaire will be refined and the correlation between the factors will be further explored to promote the effective implementation of innovation education.

## Recommendations

From the research results and findings, practical suggestions are proposed:

(1) Student entrepreneurial teams could create outcomes by the accumulation and cultivation of resources and capabilities, and the integration of resources acquired from external network combination could assist in the innovation technology results, create value, and acquire entrepreneurship outcome. For this reason, a student entrepreneurial team could collaborate with other entrepreneurial teams, enterprises, and investors to real-time share external trend at the time, dig out technology frontier and business opportunities, jointly seek for possible benefits and usage of products, and further satisfy customer needs for value innovation as well as develop innovative product markets to acquire higher business performance.
(2) A student entrepreneurial team has to constantly enhance the collaboration ability, maintain good communication models, work hard to help partners solve business problems, and further jointly develop future need planning and technology, in order to develop a tighter connection. In addition to the consolidation of original partners, a student entrepreneurial team could further attract new partners for cooperation to expand the cooperation system, enlarge the advantage of collaboration, and further enhance the innovation and entrepreneurship performance of the university student entrepreneurial team.
(3) In terms of applying the internet to execute innovation education, a student entrepreneurial team could increase the number of synchronous lessons and semi-force team members’ learning; team members’ innovative learning effect under an interactive environment would be better than a party independently accepting information. Unlike physical courses with face-to-face communication, instructors and team members or internal teams could have further communication opportunities through the right to speak or text input in network education, and the enhancement of interactivity could be achieved by the improvement of innovation education.

## Data Availability Statement

The datasets generated for this study are available on request to the corresponding author.

## Ethics Statement

The studies involving human participants were reviewed and approved by University Committee. The patients/participants provided their written informed consent to participate in this study.

## Author Contributions

DZ and YW: conceptualization. HZ: methodology. QZ: software and visualization. DZ: writing original draft preparation. YW: writing review and editing. All authors contributed to the article and approved the submitted version.

## Conflict of Interest

The authors declare that the research was conducted in the absence of any commercial or financial relationships that could be construed as a potential conflict of interest.
